# A Comparative Study among Medical Students Performing In-person Versus Virtual Workshop in Last Hours of Life Care

**DOI:** 10.1007/s40670-026-02771-y

**Published:** 2026-05-28

**Authors:** Sandra Rubio Bernabé, Leire Arbea Moreno, José Pereira, Carlos Centeno, María Arantzamendi Solabarrieta

**Affiliations:** 1 Hospital San Juan de Dios, Pamplona, Spain; 2 Faculty of Medicine, University of Navarra, Pamplona, Spain; 3 Medical Education Department, University of Navarra, Pamplona, Spain; 4 Palliative Care Department, Clinic University of Navarra, Pamplona, Spain; 5 Navarra Institute for Health Research (IdiSNA), Pamplona, Spain; 6 University of Navarra Institute for Culture and Society, ATLANTES Global Observatory of Palliative Care, Pamplona, Spain; 7 Faculty of Nursing, University of Navarra, Pamplona, Spain

**Keywords:** Palliative care, End-of-life, Undergraduate medical students, Professionalism., Last hours of life, Prospective study

## Abstract

**Background:**

Integrating palliative care into undergraduate health education is essential for preparing future professionals to care for patients facing serious illness and at the end of life. Simulation-based learning has emerged as an effective strategy to complement limited clinical exposure, particularly during the last hours of life (LHoL), a phase requiring clinical expertise and emotional sensitivity.

**Objective:**

To compare the effectiveness of in-person versus virtual simulation workshops in improving medical students’ self-perceived competency in professionalism in the context of LHoL care.

**Methods:**

A prospective, comparative pre–post study was conducted with final-year medical students at the University of Navarra. Participants were randomized to either an in-person (n=17) or virtual (n=29) simulation workshop. Both formats used standardized patients or standardized patient-based materials, structured debriefing, and targeted learning objectives. Self-perceived competencies were assessed before and after the workshop using the validated Student’s Inventory of Professionalism (SIP), which evaluates seven domains: *Holistic Care, Care and Understanding, Personal growth, Teamwork, Patient assessment, Decision-making, *and* Being a professional*. Non-parametric tests analysed within- and between-group changes.

**Results:**

Both groups showed significant improvements in overall professionalism scores post-intervention (in-person: 8.12 to 9.03, p < 0.05; virtual: 8.24 to 9.15, p < 0.05). The in-person group improved significantly across all seven domains, whereas the virtual group improved significantly in four domains but not in *Personal growth, Teamwork*, or *Being a professional*. Between-group comparisons showed no significant differences in overall improvement (p = 0.60), nor in six domains. The only exception was *Holistic Care*, where the virtual modality yielded slightly greater gains than the in-person modality (*p* = 0.04).

**Conclusion:**

Both virtual and in-person workshops enhance medical students perceived competency in professionalism. LHoL workshops provide a valuable setting to explore emotionally intense and complex scenarios that foster professional identity development. This study also highlights the value of virtual modalities in strengthening clinical reasoning and self-reflection, although their effectiveness may be reduced in areas requiring clinical immersion and collaborative skill development.

## Background

Integrating Palliative Care (PC) into the undergraduate curriculum is essential to equip medical students and learners in other health professions with the knowledge, attitudes, and skills required to care for individuals with serious illnesses, particularly at the end of life (EOL), in the context of population ageing and the increasing burden of chronic disease [1–4]. Despite its recognized importance, the European Association of Palliative Care (EAPC) has highlighted persistent gaps in training, including limited opportunities for experiential learning [4, 5]. These deficiencies are evident not only at the undergraduate level but also in postgraduate training, where variability in exposure and competencies has been reported [6, 7]. In Spain, as in many other jurisdictions, PC education remains insufficiently integrated into undergraduate health sciences curricula, often characterised by limited, heterogeneous, and predominantly theoretical teaching [2, 8–13]. Compounding this gap is a shortage of palliative care educators and experiential learning opportunities.

Various learning methods—such as lectures, case-based learning, and simulations—have been used to prepare health science students for patient care. Among these, simulation-based learning, especially with standardized patients, has become essential for developing both clinical skills and strategies to respond to emotional situations in a realistic, low-risk environment [14–16]; allowing students apply theoretical knowledge and develop critical professional skills [17, 18]. This type of experiential learning plays a key role in education by shifting learning from a theoretical to a more applied approach, promoting deeper understanding, as described in Kolb’s Experiential Learning Cycle and Miller’s Pyramid of Clinical Competence [19, 20], both of which emphasize the progressive integration of knowledge into action-based performance. The integration of experiential learning strategies is increasingly called for in palliative care training, and the use of standardized patient simulations offer such learning opportunities, especially where opportunities for bedside experiences are limited [21].

E-learning increasingly offers opportunities to enhance learning, including when experiential learning opportunities are limited [22], particularly during the COVID-19 pandemic [14], when flexible and adaptable learning formats were essential. Although terms like e-learning and virtual learning lack universally accepted definitions, e-learning broadly refers to the use of digital technologies to facilitate education. This includes both synchronous methods such as live webinars and workshops, and asynchronous tools like streaming presentations and interactive self-paced modules. Virtual learning, encompassing formats like virtual simulations and virtual reality, is considered a more immersive and interactive branch of e-learning. In palliative care education, e-learning has been adopted in both undergraduate [23] and continuing professional education [24, 25] as a strategy to address the shortage of trained educators and limited clinical training opportunities. Beyond logistical advantages, online learning modalities may, depending on design and application, enhance educational impact on domains such as communication skills, knowledge acquisition, and reflective, critical thinking [26–28]. Techniques such as scaffolding to address cognitive load management may be incorporated.

To respond to these educational challenges, the University of Navarra has developed and implemented virtual and in-person simulated scenario with standardized patients focused on care during the last hours of life (LHoL).

### Objective

The overall aim of this study was to compare the final-year medical students self- perceived level of competency in professionalism of an in-person standardized-patient workshop about LHoL care versus virtual modality. This is part of a larger mixed methods approach that included qualitative methods to supplement the quantitative methods reported in this paper.

## Materials and Methods

### Overall Study Design

A prospective comparative study with a pre-versus post design was conducted among students who participated in in-person or virtual workshop.

### Context

The standardized patient simulation workshops are part of a compulsory, 3 European Credit Transfer and Accumulation System (ECTS), palliative care course for final year medical students at the University of Navarra. The semester-long course, with over 50 study-load hours, includes lectures, case-based seminars, self-learning exercises and three hours of workshop training in a simulation centre. The latter includes a 1-hour station on subcutaneous administration of drugs and fluids, a 1-hour station on challenging conversations, and a 1-hour station on care in the LHoL.

### Intervention: Last Hours of Life Workshop

The LHoL workshop is composed of two scenes. In the first, the patient presents with terminal delirium. In the second, the patient dies while the healthcare team is present.

The workshop involves a standardized patient, portrayed by an actor, who has stage IV lung cancer and liver failure due to disease progression and who is in the final days and hours of life. He is being attended to at home by a home care team. The simulation room is assembled and decorated to resemble a home setting. The patient’s spouse, also played by an actor, is at the bedside.

In the *in-person* workshop in the simulation centre, two students are asked to volunteer to undertake the “home-visit”. They assume the roles of the physicians, accompanied by a nurse. The nurse’s role is portrayed by a real nurse who has been trained to standardize the performance. The rest of the student group observes the “visit” in real time from an adjacent room via close-circuit video. One or two teachers facilitate the workshop. Upon completing the simulation, a structured debriefing is conducted by teachers, allowing for guided reflection, feedback, and discussion of clinical and communication strategies relevant to LHoL care in PC. Two other students are then asked to volunteer for scene 2 in which a follow-up visit, the following day, is simulated; this scene too is followed by a facilitated debriefing. During scene 2, the patient “dies”, requiring the students (in their physician roles) to respond to the family member’s emotional response.

In the *virtual* workshop, the student groups (each of about 20 students as in the in-person workshop) meet live online with one or two teachers (facilitators) via the Google Meet video conferencing platform. The students are shown two trigger videos to solicit reflection and discussion [29]. Each video is about 10 min long and each one is followed by a facilitated discussion (i.e. video-discussion-video-discussion). The videos were produced by the study team and filmed in the same setting as the in-person workshop, using identical room layout and decoration. The role of doctor is portrayed by a fourth-year oncology resident, and the nurse and actors are the same as those in the in-person workshop. The strategy is based on the “Not Quite Right” approach used by Pereira et al. as part of the Learning Essential Approaches to Palliative Care (LEAP) courseware [30]. The short videos are intentionally designed to elicit reflection and discussion in the context of small group learning (in-person or in virtual webinar sessions). They show scenarios in which good, suboptimal and poor communication techniques (verbal and non-verbal) are interspersed in each video scenario. They show scenarios in which good, suboptimal and poor communication techniques (verbal and non-verbal) are interspersed in each video scenario. The design is based on the reflective learning approaches [31] which aligns with learning theories related to reflective and transformative learning [32–34].

Upon completing the workshop, the learners should be able to: [1] Understand the principles of a clinical holistic evaluation, including patient anamnesis and attending to the spouse as well, [2] identify symptoms and the fundamentals of their pharmacological management (clinical decision-making) regarding pain and other symptoms such as agitation, [3] comprehend the importance of effective communication with family members in LHoL care, [4] understand the complexity of emotional management in these situations and how to help family members cope with the loss and [5] experience the significance of self-reflection when facing complex clinical and emotional situations such as LHoL moments.

In previous years the topic of LHoL was addressed only through in-classroom didactic lectures. In the intervention studied herein, in-person and virtual experiential workshops were implemented to complement the lectures. Prior to participation in the workshops, students in both groups were provided with the same set of required pre-reading materials related to end-of-life care that included care in the last hours. This flipped classroom approach was employed to optimize student engagement and learning outcomes in both the in-person and virtual workshops. The same two teachers, both experienced palliative care clinicians and educators, facilitated both workshops across all groups, and the same actors played their respective parts for all in-person groups and the video for the virtual workshops. The principal investigator, to maintain distance and objectivity, did not participate in any teaching.

### Study Participants and Sample Size

All final-year medical students (*n* = 185) of the University of Navarra’s 2022–2023 Palliative Medicine course were invited to participate.

A sample size estimation was undertaken. Using the Students’ Inventory of Professionalism (SIP) [35] as primary outcome measure, with an alpha error set at 0.05, a difference of 17 points, and a standard deviation of 22 points, a sample estimate of 16 or 20 participants per group was determined for 80% or 90% power respectively.

### Outcome Measure

The SIP questionnaire was developed by the Palliative Care team at the University of Navarra to self-assess aspects of professionalism and has shown strong psychometric properties and validity [29]. It consists of 33 items divided into 7 domains: *Holistic care* (11 items), *Care and understanding* (6 items), *Personal growth* (4 items), *Teamwork* (3 items), *Decision-making* (6 items), *Patient assessment* (2 items), and *Being a professional* (1 item). Each item is self-scored by a student using 0 to 10 Likert-type scale (0 = completely disagree, 10 = completely agree), reflecting their attitudes and perceived learning across the domains. The instrument takes about 10 min to complete.

### Recruitment and Randomization

At the beginning of the course, students were invited by email to participate in the study. A total of 46 students volunteered to participate. Participation was entirely voluntary, and no incentives, such as extra credit or bonus points, or monetary rewards, were offered to participate. After providing informed consent, they were randomized to either the in-person workshop at the simulation center or the virtual format.

Randomization followed a stratified approach. The class is pre-divided into three large groups by the Faculty of Medicine’s administrative staff for organizational purposes. After obtaining voluntary participation, a cluster-based allocation approach was implemented using the pre-existing groups. Randomization was performed using https://echaloasuerte.com. Given the presence of three groups, allocation resulted in two groups being assigned to one modality and one group to the other; Groups 1 and 3 were randomized to the in-person workshop, while Group 2 was assigned to the virtual workshop.

### Data Collection and Analysis

Students were asked to complete the SIP immediately before and immediately after their workshop. A QR code facilitated completion of the questionnaire and 15 min were allocated for each administration (pre- and post-workshop). The responses were codified to deidentify data. The code allowed pairing of data while hiding the students’ identities from the analyst research team.

Additionally, students who attend virtual simulated scenario were asked to answer two open-ended questions, the first explored their learning experience with the workshop and the second what their key take-home messages were. The form had no word limit. This article reports only the quantitative data; the qualitative data has been reported separately.

### Data Analyses

To achieve a more homogeneous analysis, the mean score of the items within each domain was calculated for each student, resulting in an average domain score. Non-parametric statistical tests were employed, as the assumption of normality could not be guaranteed. Descriptive statistics are presented as medians and interquartile ranges (IQR) of these domain means, providing a more accurate representation of central tendency and dispersion within each group.

To analyse within-group differences (pre- vs. post-workshop) for each domain, both in the in-person and virtual workshop conditions, the Wilcoxon signed-rank test for paired samples was applied. To compare the magnitude of change between groups (in-person vs. virtual) across each domain, the Mann–Whitney U test for independent samples was used. The magnitude of change was operationalized as the difference in mean pre–post score changes between the two groups. Additionally, the Cohen’s effect size (effect r) was calculated to provide a standardized measure of the strength of the observed differences. Based on the extended interpretation of Cohen’s guidelines [36], effect sizes were classified as follows: values below 0.10 were considered negligible; values between 0.10 and 0.29 were interpreted as small; values between 0.30 and 0.49 as medium; and values equal to or greater than 0.50 were considered large.

### Ethical Considerations

The study protocol and the accompanying learning materials were reviewed and approved by the University of Navarra’s Clinical Research Ethics Committee (study 2023.035). As requested by the Ethics Committee, the LHoL workshops were assessed separately – using the instrument described herein – to the main course where students complete an end-of-course summative written multiple choice-based examination. No content covered in the LHoL workshops (virtual or in-person) was included in the main examination to avoid any potential impact on students’ grades based on workshop type. Given the emotive nature of the scenario, a psychologist was available to support students who may have experienced emotional difficulties.

## Results

A total of 185 sixth-year medical students enrolled in the Palliative Medicine course at the University of Navarra during the 2022–2023 academic year were invited to participate in the study. A total of 46 students consented to participate. Seventeen students participated in the in-class group, and all provided both pre and post workshop responses, resulting in 17 paired samples in this group. Of the 29 students who participated in the virtual workshops, only two did not complete both pre- and post-questionaries and these two were excluded from the analysis, resulting in 27 paired samples in this group.

### Baseline Data

Both groups demonstrated similar baseline scores overall, with total median scores of 8.12 for the in-person group and 8.24 for the virtual group. The difference was not statistically significant. While the virtual group showed slightly higher median scores in four out of seven domains—*Care and Understanding*,* Personal Growth*,* Teamwork*, and *Decision-Making*—these differences were modest and not statistically significant. Median scores for *Patient Assessment* and *Being a Professional* were identical across groups. *Holistic Care* was scored slightly higher on the in-person group. The virtual group also showed somewhat greater variability in some domains, as reflected by wider interquartile rates.

For the in-person workshop group, *Being a professional* and *Personal growth* are the areas with highest median scores; 9.00 and 8.50, respectively. Whereas in the virtual workshop group, *Being a professional*, *Personal growth* and *Teamwork* scored highest (9.00 for all three) (Table 1).

**Table 1:** Summary of students’ scores in the in-personal and virtual workshop Groups. ^Student Inventory of Professionalism. ‘IQR’ Interquartile range. *Z value indicates how far the observed difference deviates from the null hypothesis

Pre- workshop	In-person (*n* = 17)		Virtual (*n* = 27)			
SIP^ *domain*	Median Pre	IQR’ Pre	Pre Median	Pre IQR	*Z	p-value
*Holistic care*	7.64	0.64	7.45	1.36	-0.02	0.98
*Care and understanding*	8.33	0.83	8.67	1.50	-0.387	0.70
*Personal growth*	8.50	2.50	9.00	1.50	-0.54	0.59
*Teamwork*	8.33	2.00	9.00	1.67	-0.92	0.36
*Patient assessment*	8.33	1.17	8.33	1.34	-1.27	0.21
*Decision-making*	7.50	2.00	8.00	1.50	-0.38	0.71
*Being a professional*	9.00	2.00	9.00	1.00	-0.39	0.70
TOTAL	8.12	0.69	8.24	1.31	-0.68	0.50

Within the in-person group, the lowest median score was observed in the *Decision-making domain* (7.50), followed by *Holistic care* (7.64). In the virtual group, the domains with the lowest scores were *Holistic Care* and *Decision-making* (7.45 and 8.00, respectively).

### Comparison Pre-post In-person Workshop Group on Self-perceived Level of Professionalism

**Fig. 1:**
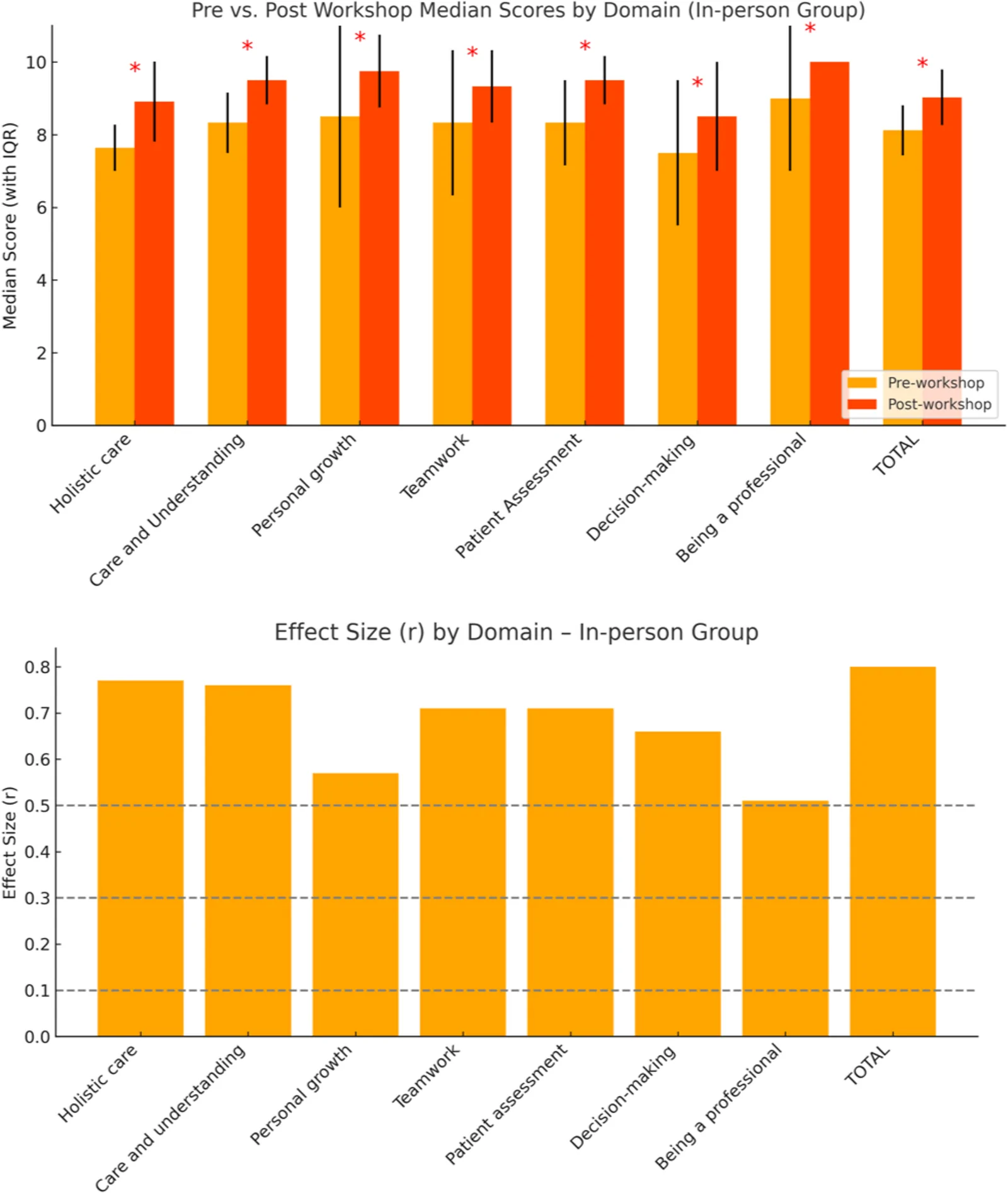
Changes in student self-perceived level of competency in professionalism following the in-person workshop. Top panel: Median scores before and after the intervention for each SIP domain. Vertical lines represent the interquartile range (IQR), and asterisks (*) indicate domains where statistically significant differences were found (*p* < 0.05). Bottom panel: Effect sizes (r) by domain, indicating the magnitude of change pre- to post-intervention. Horizontal dashed lines mark standard thresholds: values < 0.10 are negligible, 0.10–0.29 small, 0.30–0.49 medium, and ≥ 0.50 large

In the in-person group statistically significant improvements were observed in pre and post scores in all the seven domains following participation in the workshop (p-values < 0.05) and moderate to large effect sizes (r ranging from 0.51 to 0.80). There was a statistically significant increase in the total mean score from 8.12 to 9.03 (Z = -3.29, *p* = 0.001), indicating a large effect (*r* = 0.8) (Table 2) (Fig. 1).

**Table 2:** In-person workshops: Pre- versus post-workshop ^Student Inventory of Professionalism scores. IQR’ Interquartile range.*Z value indicates how far the observed difference deviates from the null hypothesis. ‘Cohen’s effect size quantifies the magnitude of the difference between groups

In-Person Workshop *n* = 17 students	Pre- workshop		Post- workshop					
SIP^ domain	Median Pre	Pre IQR	Median Post	Post IQR	*Z	p-value	‘Effect size (r)	p-value
*Holistic care*	7.64	0.64	8.91	1.10	-3.17	0.0015	0.77	0.0002
*Care and Understanding*	8.33	0.83	9.50	0.66	-3.13	0.0018	0.76	0.0003
*Personal growth*	8.50	2.50	9.75	1.00	-2.36	0.0185	0.57	0.014
*Teamwork*	8.33	2.00	9.33	1.00	-2.94	0.0033	0.71	0.001
*Patient Assessment*	8.33	1.17	9.50	0.66	-2.94	0.0033	0.71	0.001
*Decision-making*	7.50	2.00	8.50	1.50	-2.71	0.0068	0.66	0.003
*Being a professional*	9.00	2.00	10.0	0.00	-2.10	0.0361	0.51	0.031
TOTAL	8.12	0.69	9.03	0.76	-3.29	0.001	0.80	< 0.0001

In the post-intervention scores, the same pattern as in pre-test is observed: *Being a professional* and *Personal growth* are the domains with the highest median scores (10 and 9.75); and *Holistic care* and *Decision-making* have the lowest medians (8.91 and 8.50).

**Fig. 2:**
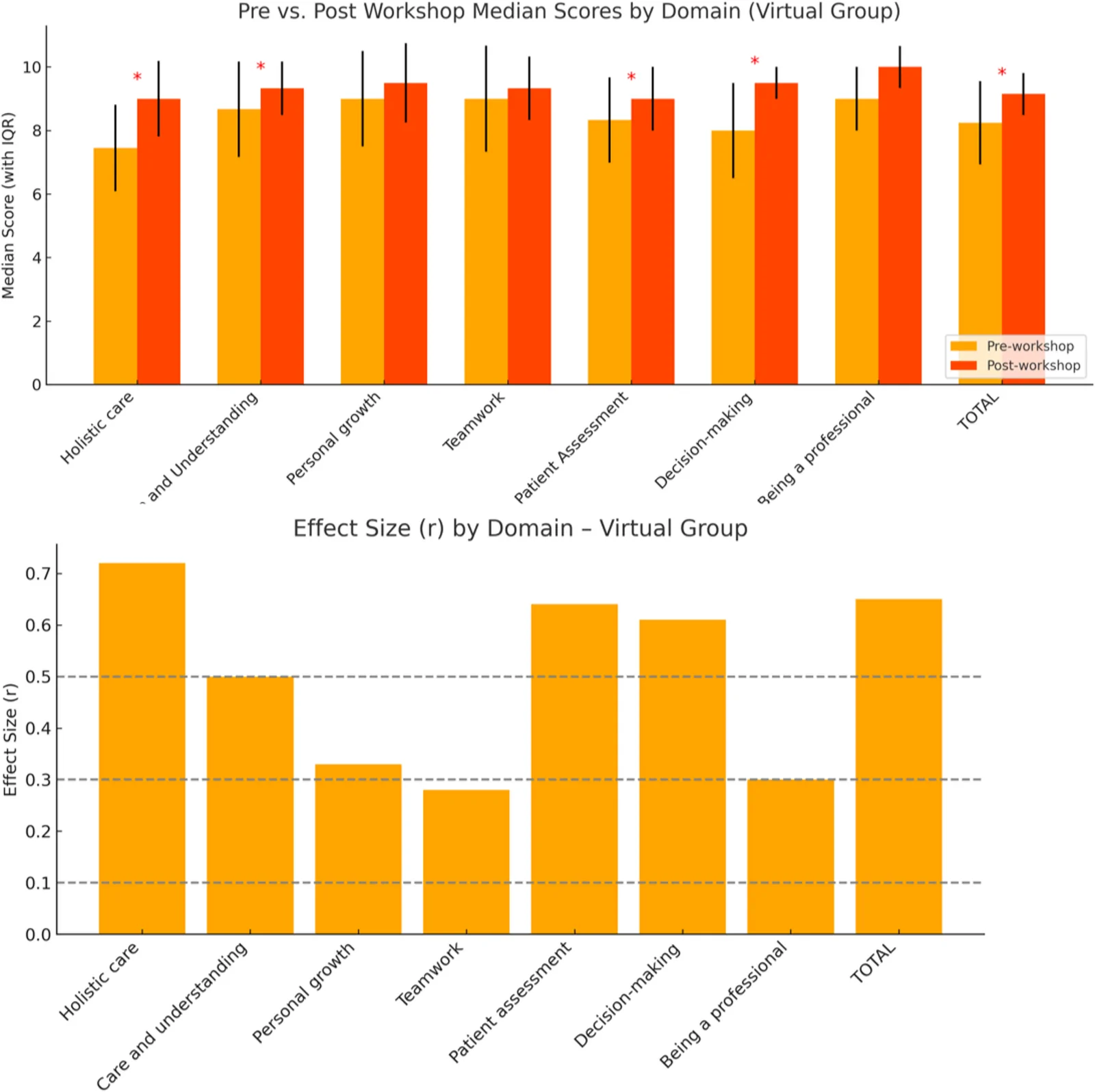
Changes in student self-perceived level of competency following the virtual workshop. Top panel: Median scores before and after the intervention for each SIP domain. Vertical lines represent the interquartile range (IQR), and asterisks (*) indicate domains where statistically significant differences were found (*p* < 0.05). Bottom panel: Effect sizes (r) by domain, indicating the magnitude of change pre- to post-intervention. Horizontal dashed lines mark standard thresholds: values < 0.10 are negligible, 0.10–0.29 small, 0.30–0.49 medium, and ≥ 0.50 large

*Comparison pre-post virtual workshop group on self- perceived level of professionalism* Overall, students reported improvements in median scores pre- versus post-workshop across all seven domains, with a statistically significant increase in the total mean score from 8.24 to 9.15 (Z = -3.36, *p* = 0.0008), indicating a large effect (*r* = 0.61) (Table 3) (Fig. 2).

**Table 3:** Virtual workshops: Pre- versus post-workshop ^Student Inventory of Professionalism scores. IQR’ Interquartile range. *Z value indicates how far the observed difference deviates from the null hypothesis. ‘Cohen’s size r quantifies the magnitude of the difference between groups

Virtual Workshop *n* = 27 students	Pre- workshop		Post- workshop					
^SIP domain	Pre Median	Pre IQR’	Post Median	Post IQR’	*Z	p-value	‘Effect size (r)	
*Holistic care*	7.45	1.36	9.00	1.19	-3.72	0.0002	0.72	< 0.0001
*Care and understanding*	8.67	1.50	9.33	0.84	-2.57	0.0101	0.50	0.008
*Personal growth*	9.00	1.50	9.50	1.25	-1.70	0.0896	0.33	0.93
*Teamwork*	9.00	1.67	9.33	1.00	-1.45	0.1480	0.28	0.156
*Patient assessment*	8.33	1.34	9.50	1.00	-3.33	0.0009	0.64	0.0003
*Decision-making*	8.00	1.50	9.00	1.50	-3.15	0.0016	0.61	0.0007
*Being a professional*	9.00	1.00	10.00	1.00	-1.77	0.0766	0.34	0.084
TOTAL	8.24	1.31	9.15	0.66	-3.36	0.0008	0.65	0.0003

The difference was statistically significant in four of the domains, namely *Holistic care*,* Care and understanding*,* Patient assessment*, and *Decision-making* (Z > -1.00 and *p* < 0.05). These four areas have the largest effect sizes (*r* = 0.72, *r* = 0.65, *r* = 0.64 and *r* = 0.50, respectively). The other three domains, *Being a professional*, *Personal growth* and *Teamwork*, improve scores but do not reach statistical significance (*p* > 0.05).

In the post-test assessment, the highest and lowest score areas are not the same as in the pre-test. *Being a professional*, *Patient assessment* and *Personal growth* demonstrated the highest scores, with mean values of 10.00 and 9.50 the last two. In contrast, *Holistic care* and *Decision-making* were the lowest-scoring domains, with median of 9.00 both.

### Comparison Between the In-person Versus Virtual Group

**Fig. 3:**
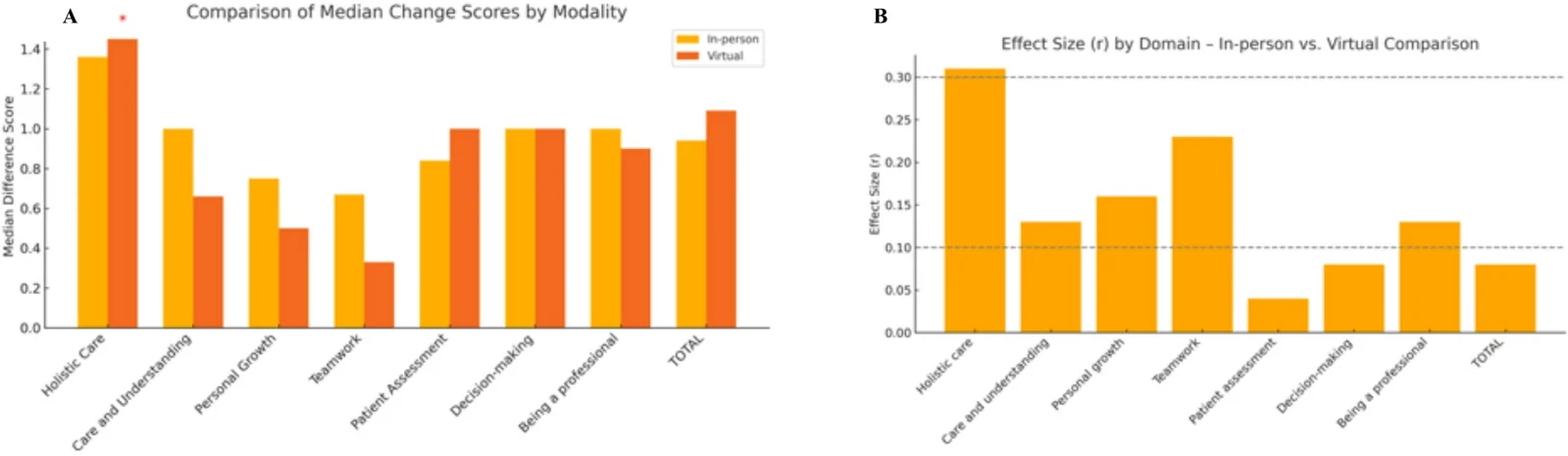
(**A**) Comparison in student self-perceived level of competency in professionalism between in-person and virtual workshop. Median scores before and after the intervention for each SIP domain. (**B**) Effect sizes (r) by domain in the comparative in person versus virtual group, indicating the magnitude of change pre- to post-intervention. Horizontal dashed lines mark standard thresholds: values < 0.10 are negligible, 0.10–0.29 small, 0.30–0.49 medium, and ≥ 0.50 large

Both the in-person and virtual workshop groups demonstrated significant improvements in the self- perceived level of competency in professionalism scores following the workshop (Tables 2 and 3). Overall, no statistically significant differences were observed in the total change scores between the two groups (Z = 0.52, *p* = 0.60), indicating that both formats produced comparable overall improvements in students’ perceived professionalism (Table 4) (Fig. 3).

**Table 4:** In-person versus virtual workshop comparison between ^Student Inventory of Professionalism domains. IQR’ Interquartile range. *Z value indicates how far the observed difference deviates from the null hypothesis. ‘Effect size r quantifies the magnitude of the difference between groups

In-Person VS Virtual	Median In-person	In-person IQR’	Median Virtual	Virtual IQR’	*Z	*p*-value	‘Effect size (*r*)
^SIP domain							
*Holistic Care*	1.36	1.0	1.45	1.82	2.04	0.04	0.31
*Care and Understanding*	1.0	0.83	0.66	1.67	0.83	0.41	0.13
*Personal Growth*	0.75	2.5	0.5	2.25	1.05	0.29	0.16
*Teamwork*	0.67	1.34	0.33	1.67	1.55	0.12	0.23
*Patient Assessment*	0.84	1.5	1.0	2.0	0.27	0.79	0.04
*Decision-making*	1.0	1.33	1.0	1.67	0.511	0.61	0.08
*Being a professional*	1.0	2	0.0	1.0	0.87	0.38	0.13
TOTAL	0.94	0.90	1.09	1.49	0.52	0.60	0.08

At the domain level, the only statistically significant difference among the two workshop modalities was in *Holistic Care* domain (Z = 2.04, *p* = 0.04). The virtual group showed a greater median change (1.45) compared to the in-person group (1.36) [Z = 2.04, *p* = 0.04, *r* = 0.31], suggesting a small to moderate effect (*r* = 0.31) in favor of the virtual format.

For all other domains, *Care and Understanding*,* Personal Development*,* Teamwork*,* Patient Assessment*,* Decision-making* and *Professionalism*, differences between groups were not statistically significant, and the effect sizes between both modalities were small or negligible (*r* < 0.1) (Table 4).

## Discussion

Both the in-person and virtual workshop groups demonstrated significant improvements in professionalism scores following the workshop. These findings suggest that both modalities may be effective in enhancing student’s self-perceived level of competency in professionalism in PC education, despite the different formats. The domains assessed by the SIP Questionnaire—*Holistic Care*,* Care and Understanding*,* Personal Growth*,* Teamwork*,* Patient Assessment*,* Decision-Making*,* and Being a professional*—are closely aligned with the learning outcomes outlined in both national and international medical education frameworks [37–41]. These competencies are explicitly included in the Spanish national curriculum [37] for undergraduate medical training, supported by national accreditation standards in PC [42], and reinforced by recommendations from leading international organizations in the PC field [43]. The workshop described in this study emerges as a valuable educational strategy for achieving key curricular goals, while also reaffirming the importance of integrating experiential and reflective learning into medical education [38, 39]. Its design not only facilitates the development of core clinical competencies but also fosters essential transversal skills such as communication, empathy, teamwork; all of which are fundamental to deliver high-quality, patient-centered care, not only at the end of life but throughout the medical profession [40]. Furthermore, the workshop contributes meaningfully to the formation of professional identity, a core curricular component consistently highlighted by global educational guidelines as essential for the development of competent and compassionate healthcare professionals [41]. Therefore, these findings may also inform curriculum design and contribute to ongoing discussions on the standardization of palliative care education in undergraduate medical training.

In the comparison between in-person and virtual modalities, the only statically significant difference between the two modalities was found in the *Holistic Care* domain, in favour of virtual modality. This domain includes essential competencies such as managing emotions, delivering bad news, demonstrating empathy, and providing support to both patients and their families. We hypothesize that in highly emotionally charged situations such as the last hours of life, the virtual approach may provide some emotional distancing for students, allowing them to consider more aspects of the interaction observed than when having to process emotional responses and feelings following the in-person encounters. The virtual modality may attenuate emotional intensity and reduce technical distractions related to the simulation, allowing students to engage in deeper reflection [26, 44]. Debriefing may also have redirected attention towards the physical, psychosocial, and spiritual dimensions of care, which are central to holistic practice. Moreover, items 7 to 11 (inclusive) of the SIP specifically explore aspects of engagement by the care provider, adequate time and attention to different aspects of the encounter and the use of silence. The video scenario used in the virtual workshops show poor application of these, thereby potentially making participants more aware of these aspects.

In contrast, other domains such as *Patient Assessment*,* Decision-Making*, and *Teamwork* are more task-oriented and grounded in observable clinical actions (i.e.: symptoms, requesting help, or adapting individualized care plans). These aspects may be less salient in a virtual environment, where the absence of direct interaction and observable errors could reduce students’ engagement with these competencies, potentially leading to conservative self-assessments. While this observation is explored in more detail in a separate qualitative study, beyond the scope of the present analysis, it nonetheless provides valuable context for interpreting these results [45].

It is also important to note that students in the virtual workshop group reported slightly higher baseline scores across most domains, although there were not statically significant. This finding may be interpreted in two ways. First, considering the self-assessment nature of the instrument, psychological factors such as social desirability bias and the psychological distance effect [46, 47] may have contributed to an overestimation of competencies. The reduced immediacy and lack of direct observation in virtual environments may favor more positive self-ratings. This could have contributed to an overestimation of competencies in the virtual group. Secondly, although participants were allocated randomly at group level, the absence of sociodemographic data limits the ability to exclude the influence of unmeasured differences (i.e.: prior experience) between the groups.

When analysed separately, the in-person group showed significant improvements across all domains, including some important competencies. The impact of in-person simulations in PC education has been reported, mainly in nursing [48–53], with more limited evidence in medical students [21, 54, 55]. Notably, a similar study conducted exclusively with nursing students [50], using an in-person simulation and a pre- and post-intervention design, reported comparable improvements in domains closely aligned with those evaluated in the present research. The impact has been noted across different competency areas, including knowledge, attitudes and skills as well as at an emotional level. In-person simulations provide an immersive experience that facilitates learning about ethical decision-making, effective communication, empathy and self-awareness, all crucial elements in PC.

In the virtual workshop group, no significant improvements were observed in *Teamwork*, *Personal growth*, and *Being a Professional*. This may be explained by the lower level of immersion and experiential learning inherent to virtual formats. For example, *Teamwork*, involves behaviors such as collaboration and requesting support, which are more readily enacted in in-person settings. This may explain the more marked improvement in this domain among participants in the in-person workshop. As for *Personal growth*, the phrasing of certain items (“My Clinical Experience”) may have disadvantaged virtual participants, who did not have direct patient contact which may have limited certain aspects of personal growth that typically emerge from hands-on exposure. Similarly, the *Being a Professional* domain may be influenced by the level of experiential engagement [17], , which emphasizes the importance of authentic experiences in the development of professional identity. Emotionally rich, hands-on experiences promote self-reflection—an essential process that may be less effectively triggered in virtual settings with limited affective engagement.

The results of this study align with some studies in which virtual simulation is used for end-of-life education [56–59]. It is acknowledged that our intervention for the virtual group was not a simulation as such, where students are immersed as active participants in a simulation scenario, but rather a process in which pre-developed videos are watched during a live followed by a facilitated webinar session that asks learners to reflect on the videos, using reflective learning techniques. Learners have evaluated the videos in the LEAP courseware as a highly useful component of the learning experience, whether delivered as in-person or live online webinar sessions [60–62].

This study has several limitations. First, the reliance on self-reported measures may have introduced bias, including social desirability and overestimation of competencies, particularly in the virtual group, potentially affecting the magnitude of the observed effects. Second, the study was conducted within a single institution, which may limit the generalizability of the results, as institutional practices, hidden curricula, teaching approaches and student characteristics may differ across settings. Third, the study was carried out within a specific educational and cultural context, which may have influenced how students perceive and self-assess domains such as communication, empathy, and holistic care.

Variability in participants’ baseline knowledge and attendance throughout the palliative care course, as well reliance on student self-rating may introduce confounders that warrant consideration when interpreting the results. Future research should incorporate objective assessment methods such as objective structured clinical examinations (OSCEs). Longitudinal studies to assess the retention and application of competencies over time is also needed.

## Conclusions

This single-institution study with an undergraduate medical education program in Spain demonstrates that virtual and in-person workshops – using reflective short videos in the virtual workshops and simulated scenarios in the in-person workshops – related to care in the last hours of life may have similar impact on aspects of professionalism as assessed by a standardized, validated self-reporting instrument. In resource-limited contexts, where infrastructure or conditions do not allow in-person simulations, the virtual approach described in this study may represent a practical and useful alternative to experiential learning methods. The study also demonstrates that end-of-life education has the potential of positive contributions not only to nurturing professionalism but also reinforce its potential to support competency-based training and meet the curricular demands of undergraduate medical education. Future research, including using objective assessment methods such as OSCEs and longitudinal designs, should be undertaken to more fully explore the role of this virtual approach compared to live simulations with standardized patients and the long-term retention of newly acquired insights and skills.
